# Treatment of Catarrhal Deafness by Electrolysis of the Eustachian Tube

**Published:** 1901-03

**Authors:** Dunbar Roy

**Affiliations:** Atlanta, Ga.


### TREATMENT OF CATARRHAL DEAFNESS BY ELECTROLYSIS OF THE EUSTACHIAN TUBE.*

BY DUNBAR ROY, A.B., M.D.,

ATLANTA, GA.

There are few chronic diseases more frequent than aural deafness. And on the other hand there are few diseases which give as much annoyance to both patient and physician as this same disease. Annoyance to the patient because of its attendant discomforts, such as noises, inability to listen to conversations, etc., and annoyance to the physician because of his most frequent inability to relieve just these symptoms. Any new method of treatment which offers hope for these afflicted mortals, or gives to the aurist ability to apply the necessary relief, is a blessing "devoutly to be wished." Deafness is a symptom and in no wise informs us from what it comes. In a previous paper I have discussed this matter fully, and therefore will not go over the same ground again. It is a well known fact to otologists that the majority of cases of progressive deafness comes from a catarrhal condition of the middle ear, eustachian tube and nasopharynx. I will throw out of consideration that form of deafness dependent upon an old suppurative process of the middle ear, which has probably destroyed the drum and ossicles, and in a great measure obliterated the windows leading into the internal ear and obtunded the sensibility of the nerve ending of the auditory nerve. Deafness dependent upon such a pathological condition gives very little chance of being benefited. But deafness, the gradually progressive form, where the drum is

*Read before Atlanta Medical Society, February 21, 1901.

retracted and thickened, where the ossicles have a certain degree of ankylosis, where the eustachian tube is narrowed through catarrhal thickening of its mucous membrane, where there is a red, irritable nasopharynx, this form is the one which can nearly always be benefited by appropriate treatment. I would leave out of consideration those forms of deafness in children dependent upon the presence of adenoids in nasopharynx, for it is too well known that all deafness will disappear after the adenoids have been removed. The central idea to be borne in mind in the successful treatment of dry catarrhal deafness is to find out thoroughly just what pathological conditions exist. The old idea of inflating the middle ear with a Politzer bag for the cure of chronic deafness is obsolete, and should be relegated to the middle ages. The futility of such a procedure can well be understood when we remember that the air passing through the Eustachian tube only temporarily affects the lumen and in no wise reduces the thickened condition of the mucous membrane. In many cases of chronic catarrhal deafness the eustachian tube is stenosed, and it is with difficulty that the air can be forced into the middle ear. Hence the tight feeling in the ears and the frequent tinnitus. On the other hand there are cases where the tube is thoroughly patulous, as for instance in the atrophic form, where the mucous membrane is more or less sclerosed. The important thing to decide is what is the condition of the lumen of the eustachian tube. Since in the large majority of cases stenosis is the rule, our endeavors will be directed to opening permanently this strictured condition. When we remember that the mucous membrane of the lumen of the eustachian tube lies practically in contact, we can readily understand how easy it is for any slight thickening of this membrane to produce a change in the atmospheric pressure within the middle ear. The effect, as we know, of such stenosis of the tube is to produce a retraction of the drum membrane through lessened internal pressure. This of course, continuing for any length of time, produces a more or less permanent retraction.

The subject of the general treatment of catarrhal deafness is too extensive to be handled in a single paper of moderate length, and my remarks this evening will be confined simply to the diagnosis and treatment of pathologic conditions of the eustachian tubes in so far as such are recognized as a cause in producing deafness.

To ascertain the condition of the lumen of the eustachian tube, whether patulous or stenosed, there are only two methods.

The first and most satisfactory method is that of *auscultation.* This consists in using the auscultation tube of rubber placed in the physician's ear and that of the patient, and then passing the air per catheter into the tympanic cavity. The physician, by constant practise, learns to determine the condition of the eustachian tube according to the sounds of the passage of air. If the tube is freely opened he can hear the air rushing in as if from an open tube. If stenosed there are various fine sibilant sounds which one would expect to find in a contracted tube. Sometimes when there is almost complete stenosis no sounds are heard. The otologist becomes familiar with these sounds just as the physician does with these sounds in the chest. Only in those cases where it is impossible to use the catheter should old methods like the Politzer bag be used for diagnostic purposes. Furthermore I hold that the middle ear should never be inflated without the use of the auscultation tube, for the sensations of the patient are too unreliable to be depended upon.

The second method is that of the passage of the eustachian bougie. This is rarely necessary for diagnostic purposes, for where the latter can be used the first method can always much more satisfactorily be applied; since probably in every eight out of ten cases of catarrhal deafness, the eustachian tube is more or less closed and our main endeavors will naturally be in trying to restore its patency. Aurists well know that even in cases where deafness is largely due to closure of the eustachian tube, that even then there are other conditions with which we must contend. Such for instance, as thickening of the drum, partial immobility of the ossicles, thickening of the openings leading into the internal ear, all of which by their presence impede very much the successful issue in treating catarrhal deafness. We also know that in those cases of deafness where there is a marked stoppage of the eustachian tube, the hearing is frequently wonderfully benefited just so soon as the caliber of the tube begins to open. For instance, we have all seen the hearing power increased several yards simply by one inflation in a patient suffering from an acute congestion of the tube.

Since in the large majority of cases of patients suffering with catarrhal deafness, the lumen of the eustachian tube is more or less closed, the endeavors of aurists have been to keep this tube thoroughly opened. Hence the main treatment of such deafness has been in the past limited almost entirely to inflations. In some cases where the condition is recent, inflations either by the Politzer or Valsalva method will accomplish good results, but in the large majority of cases we need something which will act more thoroughly upon the mucous membrane of the eustachian tube. The use of the catheter is by far the most satisfactory method, since not only can the middle ear be inflated with air, but vapors and liquid medicaments can be injected into the tympanic cavity. Then, again, it is by the conjoined use of this instrument and the auscultation tube that we diagnose the degree of patulency of eustachian tube. In the large majority of cases of catarrhal deafness we find, especially such as are accompanied by hypertrophic rhinitis or adenoid enlargement in the nasopharynx, a strictured condition of the eustachian tube. Now if this strictured condition can be removed we will have accomplished much, if not in restoration at least in a marked benefit to the hearing power of the patient. To accomplish this end we have for a long time used inflations. This does good in many cases, but often fails. Then was instituted the whalebone bougie, and gradual dilatation was produced by passing this into the tympanic cavity through the tube. In the large majority of cases this is a most admirable method, and will in the end produce a permanent dilatation of these strictures. This is readily ascertained by noting through the diagnostic tube how freely the air passes into the middle ear after the use of the bougie. Some two years ago Dr. Duel, of New York, called attention to the treatment of strictures of the eustachian tube by means of electrolysis, having devised some copper bougies of various sizes, through which the current was passed. During the last two years I have used this method in probably a dozen cases, and on the whole with most satisfactory results. It is not used in all cases of catarrhal deafness which apply to me for treatment, from the simple fact that all such cases do not have stricture of the eustachian tube. First of all, I always find out how free the lumen of the eustachian tube is by using the catheter and auscultation tube. I also, on the first examination, inject a solution of suprarenal extract into the tube in order to contract the mucous membrane, thus showing whether the stricture is true or false. The latter name I apply to those cases of stricture due to temporary swelling of the mucous membrane. Having found out what is the condition of the eustachian, I treat this with vapors and liquid medicaments for at least a week, and if there is then no improvement and the auscultation tube shows me a strictured condition, I resort to the use of rapid dilatation by means of electrolysis. I use the bougies devised by Duel, which consist of copper bougies, varying from No. 3 to No. 6 (French scale), securely mounted on No. 5 piano wire. I always start with the smallest and increase the size after a few days' treatment. The bougies are passed through the catheters until the bulbous end is just seen at the opening. The catheter is now inserted in the mouth of the eustachian tube. To the fore end of the wire standing from the catheter a handle is attached, which in turn is attached to the negative pole of a galvanic battery by means of the chord attachment. The positive pole is an ordinary sponge electrode and is held in the patient's hand. The current is now turned on until the milliamperemeter registers 5 ma. The bougie is now passed on into the lumen of the eustachian, and when it reaches the stricture I wait a few moments for the electrolytic action, and then by gentle pressure the stricture is usually passed and the bougie continued into the tympanic cavity. This latter is very necessary to learn by measuring beforehand how much of the free end of this wire must remain exposed so that the other end will be into the tympanic cavity. I use sometimes as high as 8 or 10 ma., although Duel says he never uses more than 5 ma. I always use a pure silver catheter and insulate it by wrapping with gutta-percha sheeting. The battery I use is an ordinary dry cell chloride of silver battery with 32 cells. It is absolutely necessary to have a milliamperemeter. It takes quite a little knack and practice to use successfully these bougies, for a lack of skill will frequently do harm. One precaution necessary is to never inflate an ear immediately after using the bougie for fear of emphysema. This happened to me once in my early experiences. It is remarkable sometimes to see how much benefited a patient will be after even one treatment. The hearing improves and especially is the tinnitus relieved. I have been able to benefit patients who have been treated for years by the old methods. The electrolysis is used about twice a week, and between times the mucous membrane of the eustachian tube and tympanic cavity is thoroughly medicated.

In conclusion, allow me to say that this is "no absolutely sure method," for such has as yet to be found, but the results obtained by me so far warrant the assertion that it is superior to all other treatment yet devised.

DISCUSSION.

*Dr. Wheeler:* I did not come prepared to say anything, as I came more to listen. Dr. Roy covered the ground so well that there is not much left for discussion. Dr. Roy has given a full detailed account of the method for relieving two thirds of the trouble of this nature. I have nothing to add, and I thank the doctor for his paper and the light thrown on this subject.

*Dr. J. McF. Gaston, Jr.:* In regard to the general effects of electricity, I agree with the doctor that the principles are very well established that the negative pole is effective in dissolving strictures of any kind. I have had occasion to notice this in a great many cases. For instance, stricture of the urethra will give way under it, and also in enlarged prostate we will get the same effect as described when used in the eustachian tube. Therefore, I would be prepared to indorse the treatment as mentioned by Dr. Roy. Also the description of the manner of applying it would appeal to one's judgment, and the surgical procedure in getting the tube in. The point is to reach the place of the stricture with the catheter, having the catheter insulated and passing the electrode into the tube, when the feeling of looseness as the current passes through the stricture may be observed. I would say that this should be done only by the aurist.

I have noted those same changes in the uterus mentioned by Dr.

Duncan, having under treatment now a case of hemorrhage from a tumor, and the positive pole has just the opposite effect of the negative. In this case the positive pole is used in the cervix. A month ago there was profuse hemorrhage and now there is very little, and that of a serous nature. In that connection, I should think that in adenoids that an application of the positive pole would have a tendency to coagulate the blood and disperse the growth without the use of curettes and thereby the bloody operation would be dispensed with.

In tumors we usually employ the negative pole, but in vascular tumors we use the positive pole, such as a nævus. A man now reports himself entirely well, having undergone this treatment.

The general principles of the treatment can be applied in numberless ways. I am glad to see the attempt to cure deafness, which is such a difficult thing to reach and to cure.

*Dr. Duncan:* I would like to ask the doctor if he used an anesthetic when he treated the nævus?

*Dr. Gaston:* I did not use an anesthetic, although there was some pain, but not a great deal.

*Dr. Emery:* I just want to thank the doctor for his entertaining and interesting paper, and in closing the discussion would like for him to say what action the electricity has on the tissue, as to whether it breaks the tissue down or cauterizes it? I would like for him to say something in regard to its action.

*Dr. L. B. Clarke:* I have nothing to say, except that I derived a great deal of pleasure and instruction from Dr. Roy's paper, and I am glad that I heard it.

*Dr VanGoidtsnoven:* We are never too old to learn, and I have certainly learned something to-night. I hope the paper will be published, as it will do a great deal of good. I don't know of anything harder to treat than these catarrhal forms. I have had several cases to treat in my own family. I have learned something to-night.

*Dr. Hubbard:* I want to thank Dr. Roy, in the first place, for his most excellent paper. It has taught me something. The principle is very simple, and I have in the last year or two used the electricity for the treatment of stricture of the urethra, and the same thing holds in the treatment for the eustachian tube. If you have a stricture due to a cicatricial condition, electrolysis is a good thing. I never treat these conditions in the ear, but send them to a specialist and they usually say they do nothing for them. I certainly think this treatment by electrolysis is a step in a scientific and rational way. I hope, as Dr. VanGoidtsnoven has said, that the doctor will publish this paper. It meets my views from the standpoint of a physician. I do not know much about this specialty, but from my point of view it is a rational treatment. The treatment by electricity has been decried for several years and left to the quacks, but it is now being taken up by some of our best men. Of course such a thing usually meets with some opposition to start with; everything that has had any merit has had opposition to start with.

Dr. Roy mentions that you should use a milliamperemeter. I think no man should use electricity without this. To do so is as unscientific and unrational as giving a dose of medicine without knowing how much is being given.

*Dr. Roy:* Some of the points that I want more especially to bring out are just along the line of what Dr. Hubbard has just said, and that is the fact that in the majority of cases the men do not go to the bottom of cases to find out what is the matter. These cases go to quacks, not that the doctor cannot do better, but in the rush of cases does not go to the bottom of it.

I do not use the Politzer bag, but use the vapor comminuter with compressed air, and I do not pass a catheter into the eustachian tube without using the auscultation tube, because the sensation of the patient is sometimes false and they do not know whether the air is going into the ear or not, but by using the auscultation tube you can tell positively yourself.

In regard to adenoids, I am sorry to hear the doctor mention the treatment he does. I think it is entirely out of place to attempt to reduce adenoids with a positive pole, for the simple reason that they are like enlarged tonsils and have to be removed surgically. In regard to the removal of adenoids, I have almost reached the point of advocating that every child should be examined in the nasopharyngeal space, and that adenoids should be removed as early as possible. It seems to me that almost nine out of ten children have adenoids, and such can be removed at almost any age, and the earlier the better. I have removed them as early as six and twelve months of age. At twelve months they are sufficiently old for the removal of these growths, and if thoroughly removed at this time with an anesthetic the child will not probably be troubled with deafness, because you remove the cause of the trouble. Adenoids should always be suspected when a child breathes through the mouth continuously.

As to what change is produced by the action of the electric current, there is an increased flow of blood and a stimulation of all the cells of the tissue and an absorption of the exudate at that point.

Before I close, I have specimens of two interesting cases. One was from a negro brought into the hospital and who had been in a brawl one night and was struck in the eye, but with what he did not know. He was brought into the hospital unconscious and had immense edema of the upper and lower lids and ecchymosis of the conjunctiva. Upon examination, no foreign body could be seen in the eye, but there was evidently some rupture of the globe. At that time I thought that if the edema went down and there was no infection, he could at least retain the form of the globe. But in 36 hours the wound became infected and panophthalmitis developed and suppuration set in. The cornea appeared perfectly clear and continued so for three or four days. About a week after the injury (not having been to the hospital for two or three days and in the meantime cold applications to the eye having been kept up, together with protargol for the suppurative condition), as I opened the lids I thought I saw a dark pupil shining through the cornea, and yet it looked out of place, and so I called for a probe, and I found a little hard mass which I removed with a pair of forceps, and which I have here. This had been embedded in the back part of the globe and had evidently been worked forward by the suppurative process. As soon as this was removed the edema rapidly subsided in twenty-four hours and he made a rapid recovery, but of course the globe was shrunken.

Another case had ectropion of the lower lid, and there seemed to be a fistulous tract and some old cicatricial tissue turned in. It felt as if there was some necrosed bone. The cicatricial tissue pulled the lid down, producing the ectropion. I cocainized the parts and was going to do an operation by freeing the lid, and in going down I found something hard which I thought to be a grain of dirt. I took a pair of forceps and drew out this piece of knife blade. He gave a history of having been struck with a knife over thirty years ago, and that the scar had been there ever since, and that it had been discharging at times. As soon as this was removed, I took out the cicatricial tissue with complete recovery from the ectropion.

---

### INJURIES TO THE WRIST.

The landmarks of the wrist, observes Edward A. Tracy (*Virginia Medical Semi-Monthly,* Nov. 9) are the styloid processes of the radius and ulna. The radial process is normally a trifle lower than the ulnar. When this relative position is reversed, fracture is to be diagnosed, whereas if the styloids are in normal relations with the wrist we have a sprain to deal with. A severe sprain is accompanied by tearing of the surrounding tissues (manifested by ecchymosis) and by tenosynovitis, characterized by a rubbing crepitus, felt by placing a hand over them while in action. Such sprains are rare without accompanying fracture of the lower end of the radius, and they should be treated as fractures. Treatment in general of wrist sprains should comprise rest to the injured joint, means to cause absorption of the tissue exudates, and means to prevent pathological adhesions. The first indication is well met by cutting a piece of wood-plastic splint material, moistening it with water, then moulding and bandaging it over the back of the forearm and hand without cotton batting or other padding. Careful massage is the best mothod of causing absorption. One should gently grasp and squeeze the forearm a little above the swelling (towards the elbow), then relax the grasp and squeeze about a half-inch below, continuing thus until we reach the lower limit of the swollen tissues—then a swift, gentle, gliding pressure from below upwards should be done. Passive motion, properly applied, prevents adhesions. The fingers should be bent daily, and the wrist about the fifth day, gradually at first. The splint above described permits readily of these movements.

